# Supplementary material for: Covid19: Unless one gets everyone to act, policies may be ineffective or even backfire
Source: PLoS One. 2020 Sep 11;15(9):e0237057. doi: 10.1371/journal.pone.0237057 (PMC7486132; doi:10.1371/journal.pone.0237057)
Supplement: S1 Document — (PDF) [file pone.0237057.s001.pdf]

PLOS One Supporting Information

Article title: Covid19: unless one gets everyone to act, policies may be ineffective or even backfire

Authors: Alessio Muscillo[1], Paolo Pin[1,2*], Tiziano Razzolini[1,3],

1 Dept. Economics and Statistics, University of Siena, Siena, Italy
2 IGIER & BIDSA, Bocconi University, Milan, Italy
3 IZA, Bonn, Germany

* paolo.pin@unisi.it

The following supporting information is available for this article:

Empirical Analysis

In this section we describe how we collected the data used for the empirical analysis, which are of two types: data on the number of contacts and data on the number of confirmed cases and deaths.

Data on number of contacts

The data on regional average number of contacts and their variance are constructed using the public dataset provided by Belot and colleagues [1]. This survey contains question on the number of contacts "on a typical working day (before the outbreak of Covid-19)" and "on a typical day in the last 2 weeks". The total number of contacts of each individual has been constructed using the number of contacts for more than 15 minutes with any person (children, adult or elderly people). The variables used are listed in Table 1 and some descriptive statistics are shown in Table 2.

Table 1. Variables used from survey.

contacts before outbreak	contacts in last 2 weeks before interview
`close_workint_more_child`	`close_recentint_more_child`
`close_workint_more_adult`	`close_recentint_more_adult`
`close_workint_more_elder`	`close_recentint_more_elder`

We have dropped 3 outliers (out of 13,023 observations in the Italian, British and South Korean regions) that is individuals with more than 800 contacts, in the pre- and post-outbreak period. Notice that 800 corresponds to twenty times the 95th percentile and more than 5 times the 99th percentile of the distribution contacts in the pre-outbreak period.

Data on number of cases and deaths

Information on number of confirmed cases and number of death has been collected from publicly available datasets. Some descriptive statistics are shown in Table 2.

- Data for Italy and South Korea are collected as follows.

Table 2. Descriptive statistics.

	Pre-outbreak	Post-outbreak (last 2 weeks before interview)	Δ (difference post-pre)
Confirmed Cases	299.625<br>(648.974)	812.188<br>(1244.210)	512.563<br>(1005.220)
Deaths	9.875<br>(35.830)	212.792<br>(328.062)	202.917<br>(305.180)
$\langle d \rangle$	8.270<br>(4.991)	3.949<br>(2.400)	-4.321<br>(5.006)
$\langle d^2 \rangle$	1175.600<br>(1747.951)	194.638<br>(361.876)	-980.962<br>(1629.347)
N. obs.	48	48	48

Mean and standard deviation (in parantheses) of the variables for the 48 regions in the sample. $\langle d \rangle$ is the average number of contacts in each region. $\langle d^2 \rangle$ is the average squared number of contacts in each region.

- Information for Italian regions (confirmed cases and deaths) are provided by *Protezione Civile* and available at COVID-19 Italia - Monitoraggio situazione.
- Information for South Korean regions (confirmed cases and deaths) are available at Data Science for COVID-19 (DS4C).
- For both Italian and South Korean regions, the weekly number of confirmed cases and deaths (1-8 March, 13-29 April) have been computed from the corresponding daily cumulative numbers.

- Data for the United Kingdom are collected as follows.
  - Data on the number of deaths for Wales and the regions of England are provided on a weekly basis (from Friday to Friday) and available from the Office for National Statistics.
  - Data on the cumulative daily number of cases in the regions of England are available at Coronavirus (COVID-19) in the UK. The weekly number of cases is computed from the cumulative daily cases for the week 13-20 March and the week from April 24 to May 1, to have this measure on the same time window used by death weekly data.
  - Weekly data on confirmed cases for Wales are computed from daily data and are available at Public Health Wales Health Protection.
  - Weekly data on deaths and cases for Northern Ireland are computed from cumulative daily data and are available at Northern Ireland Department of Health coronavirus information.
  - Weekly data on deaths and cases for Scotland are computed from cumulative daily data and are available at Scottish Government coronavirus information.

In Fig 1 we plot the pairwise relations among the variables used for the empirical analysis, for the countries considered (Italy, South Korea and the U.K.), where each point is a region of a country. These plots show the high variability in these variables, even if the three countries have been hit by Covid19 in the same period.

Robustness check

In Table 3 we investigate the presence on a non-linear relation between the the number of cases and deaths and the average number of contacts. The inclusion of the square of

Fig 1. Pairwise relations among the variables considered.

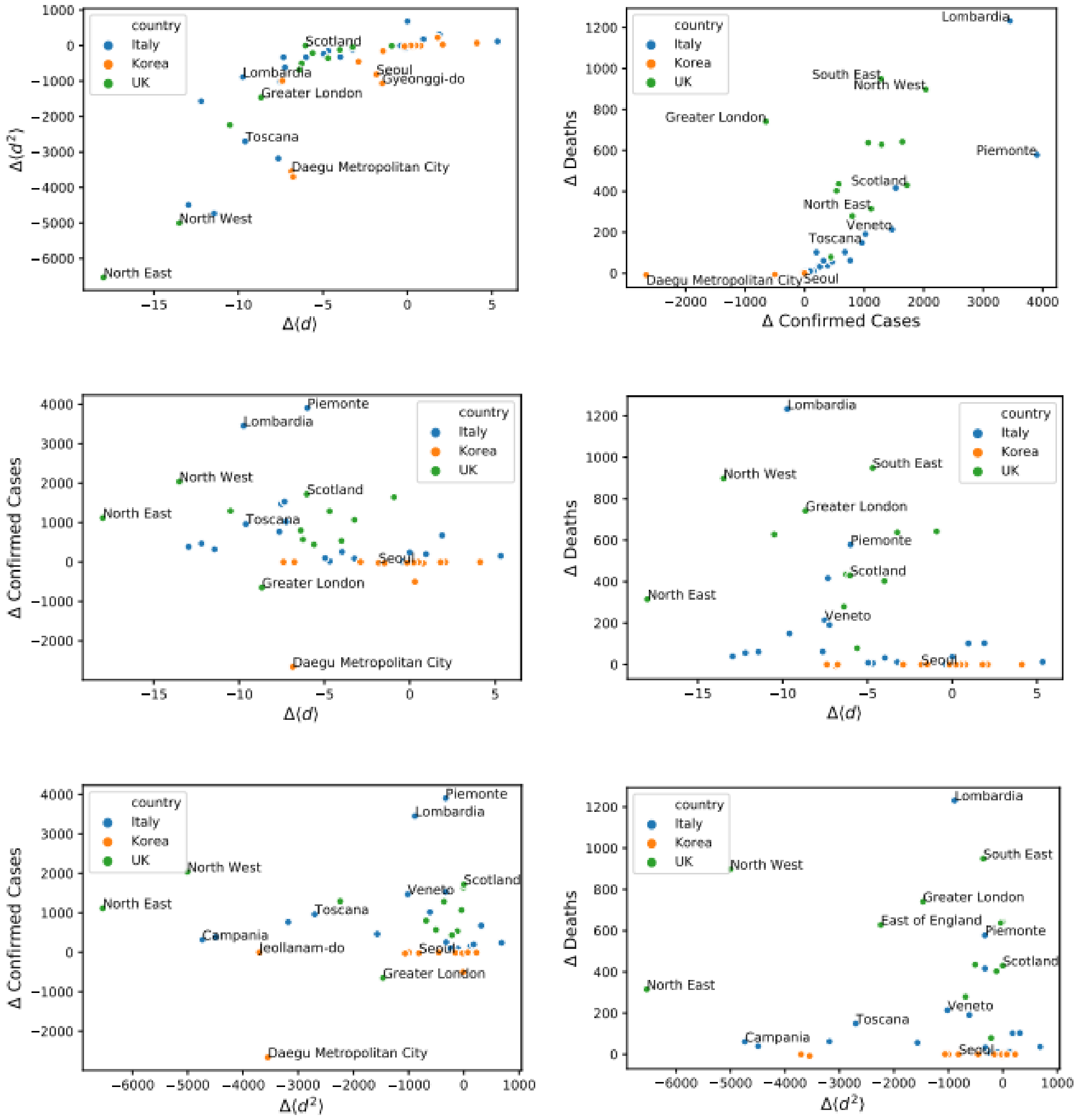

the average number of contact does not alter the qualitative impact of $\Delta\langle d^2\rangle$ nor its statistical significance.

The model

Consider a society formed by a large number of individuals who interact by meeting others at random and where each individual can alternate between being susceptible or infected to a disease which transmits via social contacts. More in detail, we first consider a degree-based random mixing model with an infinite number of agents, often thought as an "approximation" of a large social network. Then, we consider a tractable first approximation of a susceptible-infected-susceptible (SIS) model based on a simple linear form, standard in the literature [3, 4]. Although we are not the first ones to

Table 3. Variations in cases and deaths.

	Δ Confirmed Cases		Δ Deaths	
	(1)	(2)	(3)	(4)
$\Delta\langle d\rangle$	-67.945	-67.599	-27.361	-27.275
	(64.513)	(61.189)	(19.401)	(19.083)
$\Delta\langle d\rangle^2$	-0.067	-7.971*	0.094	-1.862
	(3.113)	(4.429)	(1.148)	(1.328)
$\Delta\langle d^2\rangle$		0.588**		0.146***
		(0.219)		(0.048)
Constant	214.174**	227.193**	91.448***	94.671***
	(89.785)	(98.383)	(31.568)	(32.896)
N. obs.	48	48	48	48
Adj. R^2	0.080	0.297	0.139	0.278

In columns 1 to 2 the dependent variable is the variation in the numbers of confirmed cases in a region. In columns 3 to 4 the dependent variable is the variation in the number of deaths in each region. $\Delta\langle d\rangle$ is the variation in the average number of contacts in each region. $\Delta\langle d^2\rangle$ is the variation in the average squared number of contacts in each region. $\Delta\langle d\rangle^2$ is the variation in the square of average number of contacts in each region. *** significant at 1%, ** significant at 5%, * significant at 10%.

consider this model in a context of quarantine, all previous works have focused on different issues and did not consider the possible negative effects of a quarantine that is not homogeneous [5–7]. Technically, we adopt a SIS model because its ergodic nature delivers neat analytical results. Moreover, it is still not clear to scientists whether Covid-19 can affect more than once the same person. Cases of multiple infection in the same person have been reported by the end of February in China and Japan.

Consider a network with degree distribution $P(d)$, i.e. where the degree of a node i is d_i and $P(d)$ is the fraction of individuals with degree d. The probability of meeting an agent of degree d is $P(d)d/\langle d\rangle$. Let $\rho(d)$ be the fraction of individuals of degree d who are currently infected, so that the probability of meeting an infected agent of degree d is

$$\rho(d)\frac{P(d)d}{\langle d\rangle}.$$

Overall, the probability of meeting an infected individual is

$$\theta = \sum_d \rho(d)\frac{P(d)d}{\langle d\rangle}, \tag{1}$$

while the average infection rate in the population is $\rho = \sum_d \rho(d)P(d)$.

The mechanism of the disease transmission is as follows. The chance that a given individual of degree d becomes infected in a given period when faced with a probability θ that any given meeting is with an infected individual is

$$\beta\theta d,$$

where $\beta \in (0,1)$ is a parameter describing the rate of transmission of the infection in a given period. The probability that an infected individual recovers (and becomes again susceptible) in a given period is $\delta \in (0,1)$.

With a mean-field approach, one can compute the expected change of $\rho(d)$ over time, for all d

$$\frac{\mathrm{d}\rho(d)}{\mathrm{d}t} = (1-\rho(d))\beta\theta d - \rho(d)\delta, \tag{2}$$

where the first term describes the inflow of susceptibles becoming infected and the second term describing the outflow, i.e. infected who recover.

The steady-state of the system is such that $\mathrm{d}\rho(d)/\mathrm{d}t = 0$. Solving this equation yields

$$\rho(d) = \frac{\lambda\theta d}{\lambda\theta d + 1}, \tag{3}$$

where $\lambda := \beta/\delta$. Plugging Eq. (3) into Eq. (1) gives the condition

$$\theta = \underbrace{\frac{1}{\langle d\rangle}\sum_d \frac{\lambda\theta d^2 P(d)}{\lambda\theta d + 1}}_{=:H(\theta)}. \tag{4}$$

The function $H(\theta)$ keeps track of how many individuals would become infected starting from a level θ. Steady states of the system are fixed points such that $H(\theta) = \theta$ and Eq. (4) has always solution $\theta = 0$, but can also have other solutions. Since $H(0) = 0$ and $H(\theta)$ is increasing and strictly concave in θ, then it turns out that in order to have a (unique) positive steady state it must be that $H'(\theta) > 1$. Since $H'(\theta) = \lambda\langle d^2\rangle/\langle d\rangle$, then the condition for an endemic equilibrium to exist is (and corresponding also to a positive average infection rate in the population, $\rho > 0$)

$$\lambda > \underbrace{\frac{\langle d\rangle}{\langle d^2\rangle}}_{=:\mu}. \tag{5}$$

This condition means that the infection-to-recovery ratio has to be high enough relative to average degree divided by second moment (roughly variance of degree distribution). Intuitively, this shows that high degree nodes are more prone to infection and, since they have many meeting, also serve as conduits for infection. In general, a social network with high variance in the degree distribution is such that there are many of such high degree nodes.

Endemic Disease from Self-isolation

From Eq. (5), we have that if $\mu = \langle d\rangle/\langle d^2\rangle$ decreases, then the epidemics can become endemic. This can happen, for example, if during a self-isolation period only the nodes with low degree reduce drastically their contacts.

In general, consider the situation in which all nodes decrease their contacts by a common discrete number h, obtaining a new re-scaled degree distribution $\hat{d} = d - h$. The mean degree becomes $\langle\hat{d}\rangle = \langle d\rangle - h$, but the variance of the degree distribution $\langle\hat{d}^2\rangle - \langle\hat{d}\rangle^2 = \langle d^2\rangle - \langle d\rangle^2$ remains unchanged. However, this new distribution is such that the threshold $\langle\hat{d}\rangle/\langle\hat{d}^2\rangle$ in Eq. (5) is:

$$\frac{\langle d - h\rangle}{\langle (d-h)^2\rangle} = \underbrace{\frac{\langle d\rangle - h}{\langle d^2\rangle - 2h\langle d\rangle + h^2}}_{=:\mu(h)}.$$

Since

$$\left.\frac{\mathrm{d}\mu}{\mathrm{d}h}\right|_{h=0} = \frac{-\langle d^2\rangle + 2\langle d\rangle^2}{\langle d^2\rangle^2},$$

then it is negative when $\langle d^2\rangle > 2\langle d\rangle^2$, which holds if the standard deviation is high enough. For h small, this marginal effect remains negative which indicates that $\mu(h)$ decreases. Specifically, as h increases then $\mu(h)$ decreases as long as h does not exceed

$$2\langle d\rangle - \frac{\langle d^2\rangle}{\langle d\rangle}.$$

This implies that if the cut to links imposed by the self-isolation policy is too weak, i.e. h is too small, then the threshold for the existence of the endemic equilibrium decreases. Thus, a disease that was not endemic may instead become endemic.

Speed of Recovery to Disease-free Equilibrium

From Eq. (2) we can compute the Jacobian J when the disease is not endemic. That is, when $\rho(d) \to 0$ for all d, and also $\theta \to 0$. Deriving Eq. (2) with $\rho = 0$ and $\theta = 0$ yields

$$J_{k\ell} = \begin{cases} \frac{\beta}{\langle d \rangle} k^2 P(k) - \delta, & \text{if } k = \ell, \\ \frac{\beta}{\langle d \rangle} k\ell P(\ell), & \text{if } k \neq \ell, \end{cases}$$

which can be written as the $(D \times D)$-matrix $J = (J_{k\ell})_{k,\ell=1,\ldots,D}$ of the form (where D is the maximum degree in the network)

$$J = \frac{\beta}{\langle d \rangle} \begin{pmatrix} 1 \\ \vdots \\ k \\ \vdots \\ D \end{pmatrix} \big(1P(1) \cdots \ell P(\ell) \cdots DP(D)\big) - \delta I,$$

where in the first term there is the matrix multiplication between two vectors and I is the identity matrix.

In general, consider a matrix $A := uv' - \delta I$. Then, its eigenvalues are $-\delta$ and $v'u - \delta$. The corresponding eigenvectors are, respectively, all vectors orthogonal to v and u itself. In our case, then, the only eigenvalues of J are

$$e_1 = -\delta \qquad \text{and} \qquad e_2 = \beta \frac{\langle d^2 \rangle}{\langle d \rangle} - \delta \equiv \frac{\beta}{\mu} - \delta.$$

While $e_1 = -\delta$ is independent of the network and always negative, e_2, which is proportional to the difference $1/\mu - 1/\lambda$, is negative if and only if $\lambda < \mu$. From Eq. (5), this occurs exactly when the only equilibrium is the disease-free equilibrium and it is asymptotically stable.

Moreover, from the policy perspective, in this case the speed of convergence to the disease-free equilibrium is determined by

$$|e_2| = \delta - \frac{\beta}{\mu}.$$

This implies that as $\mu = \langle d \rangle / \langle d^2 \rangle$ increases, so does $|e_2|$ and the speed of convergence to the disease-free equilibrium increases as well. Conversely, as μ decreases, so does the speed of convergence, up to the point where μ goes below the threshold λ in Eq. (5), which is when the equilibrium becomes endemic.

Stata code for data analysis

File S1. STATA code for data analysis.

Python code for figures

File S2. Python code for figures.

References

1. Michèle Belot, Syngjoo Choi, Julian C Jamison, Nicholas W Papageorge, Egon Tripodi, and Eline van den Broek-Altenburg. Six-country survey on Covid-19. 2020.

2. Thomas Hale, Sam Webster, Anna Petherick, Toby Phillips, and Beatriz Kira. Oxford Covid-19 Government Response Tracker, Blavatnik School of Government. *Data use policy: Creative Commons Attribution CC BY standard*, 2020.

3. Romualdo Pastor-Satorras and Alessandro Vespignani. Epidemic dynamics and endemic states in complex networks. *Physical Review E*, 63(6):066117, 2001.

4. Matthew O Jackson. *Social and Economic Networks*. Princeton University Press, 2010.

5. C Lagorio, Mark Dickison, F Vazquez, et al. Quarantine-generated phase transition in epidemic spreading. *Physical Review E*, 83(2):026102, 2011.

6. Huiyan Kang, Kaihui Liu, and Xinchu Fu. Dynamics of an epidemic model with quarantine on scale-free networks. *Physics Letters A*, 381(47):3945–3951, 2017.

7. Kezan Li, Guanghu Zhu, Zhongjun Ma, and Lijuan Chen. Dynamic stability of an SIQS epidemic network and its optimal control. *Communications in Nonlinear Science and Numerical Simulation*, 66:84–95, 2019.

8. Roy M Anderson, Hans Heesterbeek, Don Klinkenberg, and T Déirdre Hollingsworth. How will country-based mitigation measures influence the course of the COVID-19 epidemic? *The Lancet*, 2020.

9. Joel R Koo, Alex R Cook, Minah Park, Yinxiaohe Sun, Haoyang Sun, Jue Tao Lim, Clarence Tam, and Borame L Dickens. Interventions to mitigate early spread of SARS-CoV-2 in Singapore: a modelling study. *The Lancet*, 2020.

10. Matteo Chinazzi, Jessica T Davis, Marco Ajelli, et al. The effect of travel restrictions on the spread of the 2019 novel coronavirus (COVID-19) outbreak. *Science*, 2020.

11. Yu-Tao Xiang, Yu Jin, and Teris Cheung. Joint international collaboration to combat mental health challenges during the coronavirus disease 2019 pandemic. *JAMA psychiatry*, 2020.

12. Sandro Galea, Raina M Merchant, and Nicole Lurie. The mental health consequences of Covid-19 and physical distancing: The need for prevention and early intervention. *JAMA internal medicine*, 2020.

13. Betty Pfefferbaum and Carol S North. Mental health and the Covid-19 pandemic. *New England Journal of Medicine*, 2020.
