# Supplementary material for: Covid19: Unless one gets everyone to act, policies may be ineffective or even backfire
Source: PLoS One. 2020 Sep 11;15(9):e0237057. doi: 10.1371/journal.pone.0237057 (PMC7486132; doi:10.1371/journal.pone.0237057)
Supplement: Attachment — Submitted filename: reply_referees.pdf [file pone.0237057.s004.pdf]

Reply to Editor and Reviewers for PLOS ONE: PONE-D-20-09143: *Covid19: unless one gets everyone to act, policies may be ineffective or even backfire*

Alessio Muscillo[a], Paolo Pin[a,b], and Tiziano Razzolini[a,c]

[a]Department of Economics and Statistics, University of Siena
[b]IGIER and BIDSA, Bocconi University
[c]IZA and LABOR

June 2020

General Replies

We first of all would like to thank the editor and the three reviewers for their excellent and precious comments.

In this document, we rewrite all your comments, and provide in blue our answers. **At this link**, you can find a version of the revised manuscript that is the same one that we have resubmitted, but where all the new parts are highlighted in a different color for readability.

Following the advice of the referees, we have revised the paper in two ways:

- first, we have added an empirical motivation, using data from the survey of Belot et al. (2020) – this exercise shows that the second moment of the distribution of contacts is fundamental in predicting the actual diffusion of Covid19 in the UK, Italy and South Korea (coherently with the theoretical model);
- second, we have edited language and style, dividing also the manuscript in sections.

In order to fulfill the data analysis we have needed the help from Tiziano Razzolini, a colleague from our Department that has the experience in econometric analysis that we (Alessio and Paolo) do not have. We hope it is not a problem if we include Tiziano among the coauthors of this paper.

Reviewer 1

In this paper the authors discuss the impact of COVID-19 spreading on social groups, focusing on those who are heterogeneous in terms of social interactions (people with few and people who have more social relations and interactions). To this end, they proposed a susceptible-infected-susceptible SIS model, considering features such as transmission and recovery rates of a disease and the contact numbers of individuals.

As conclusion, the authors argue that when a disease spreads in a population with heterogeneous levels of social meetings, if the individuals who meet many people don't fully respect isolation policies, such policies can be ineffective or damaging.

The study is interesting and could help governments and health authorities to propose more efficient social isolation policies. I agree that if not everyone does their part by maintaining social isolation as long as possible, the disease may remain active for more time. However, when considering the pandemic caused by Corona virus, even if not all people reduce social interactions to the minimum possible, that still may contributes to reducing the number of patients simultaneously infected, preventing collapses in health systems.

While the study appears to be sound, I miss some validation of the model. Would it be possible to include in the article some data related to the spread of COVID-19 observed in some countries, such as the contagion rate, in contrast to the different rates of social isolation in each of them? That would be nice and could corroborate the conclusion of the study.

The authors mentioned some related work (references 6-8). I suggest including a brief description of the related work. Even though the authors claim that the proposed model is different and consider other features, that description could [...][1]

I also suggest, if possible, to compare the proposed model with the others, allowing to assess the impact of heterogeneous quarantine, once that feature was not considered in the related.

Answer: We thank the referee for the comments, upon which we agree. As discussed in the general replies above, we have included a section with an empirical motivation, right after the introduction. This exercise goes exactly in the direction predicted by our model.

In relation to other objectives that a policy maker could have, with respect to the diffusion of Covid19, we have added a discussion at the end of the main part of the paper where we acknowledge the limitations and the specific focus of our approach. We also admit that other aspects may play a role in the trade-off between lockdown measures and re-opening policies.

[1] This part was missing in the email we received from the editor.

Review 2

This article is on a feature that must be highly considered in the context of pandemic COVID-19/SARS-COV2, which regards social/physical distancing/isolation to slow the spread of the infection, avoid the collapse of health systems and buy some time for researchers come up with specific, effective treatment and/or vaccine. Since there is evidence toward distancing/isolation to slow the spread of a highly contagious disease, it seems this article boosts the recommendation of such measure and provides endorsing mathematical and simulation models. There is ongoing discussion, though, on the mental health consequences of distancing/isolation which is inevitable and should also be considered, and that health care systems/policies and providers should prepare to minimize its impact on the population, as discussed in JAMA (Xiang et al., 2020; Galea et al., 2020) and NEJM (Pfefferbaum and North, 2020).

Answer: We thank the referee for the comments and the suggested references. The point is related to what written by Referee 1 about policies. In relation to other objectives that a policy maker should have with respect to the diffusion of Covid19, we have added a discussion (and added the references) at the end of the main part of the paper, where we admit that our model is focused only on one specific aspect, while others may have a role in the trade-off between lockdown measures and re-opening policies.

Reviewer 3

The manuscript provides an innovative approach using a susceptible-infected-susceptible (SIS) model to advocate a "proportional reduction" on social contacts in order to contain new SARS-Cov-2 new infections on populations. The authors proposed a parsimonious SIS models to sustain that when a disease spreads in a given population with heterogeneous intensity of meetings, policies advocating homogeneous social contacts may be ineffective and even deleterious.

The manuscript contains some colloquial expressions and statements such as: "In a nutshell, unless one gets everyone to act, and specifically those who have more contacts, a policy may even be counterproductive". The conclusions of the paper seems a little vague: "The specific focus of our approach is to distinguish people by their degree of socialization, and remark that if not everybody reduces drastically and proportionally their social contacts, then such measures could have an effect opposite to the one expected."

The authors state that previous published works have focused on models in a context of quarantine bud did not consider the "possible negative effects" caused by the heterogeneity of the quarantine, bud provide few empirical data to sustain such a statement.

There's no limitation section on the manuscript describing the potential limitations and pitfalls of the provided model. The appendix provide the mathematical notation of the model and its applications but again, providing no empirical data. Although one can argue in defense of such a statistical note, it is worth considering that this approach may not be exactly of interest to the Plos One's audience.

Answer: We thank the referee for the comments and suggestions and, indeed, we have included a section with an empirical motivation to sustain our argumentation, right after the introduction. This exercise goes exactly in the direction predicted by our model.

We have also edited the paper to make it more formal, more focused, and updated to the present worldwide situation of Covid19.

References

Belot, M., S. Choi, J. C. Jamison, N. W. Papageorge, E. Tripodi, and E. van den Broek-Altenburg (2020). Six-country survey on Covid-19.

Galea, S., R. M. Merchant, and N. Lurie (2020). The mental health consequences of covid-19 and physical distancing: The need for prevention and early intervention. *JAMA internal medicine*.

Pfefferbaum, B. and C. S. North (2020). Mental health and the covid-19 pandemic. *New England Journal of Medicine*.

Xiang, Y.-T., Y. Jin, and T. Cheung (2020). Joint international collaboration to combat mental health challenges during the coronavirus disease 2019 pandemic. *JAMA psychiatry*.
